# Expression of microRNA-454 in TGF-β1-stimulated hepatic stellate cells and in mouse livers infected with *Schistosoma japonicum*

**DOI:** 10.1186/1756-3305-7-148

**Published:** 2014-03-31

**Authors:** Dandan Zhu, Xue He, Yinong Duan, Jinling Chen, Jianxin Wang, Xiaolei Sun, Hongyan Qian, Jinrong Feng, Wei Sun, Feifan Xu, Lingbo Zhang

**Affiliations:** 1 Department of Pathogen Biology, School of Medicine, Nantong University, 19 Qixiu Road, Nantong, Jiangsu 226001, People’s Republic of China; 2 Department of Clinical Laboratory Medicine Center, Affiliated Hospital of Nantong University, Nantong, Jiangsu Province 226001, China; 3 Cancer Research Center, Affiliated Tumor Hospital of Nantong University, Nantong, Jiangsu Province 226001, China

**Keywords:** miR-454, TGF-β1, Hepatic stellate cell, Hepatic fibrosis, *Schistosoma japonicum*

## Abstract

**Background:**

In the process of hepatic fibrosis, hepatic stellate cells (HSCs) can be activated by many inflammatory cytokines. The transforming growth factor-β1 (TGF-β1) is one of the main profibrogenic mediators. Recently, some studies have also shown that microRNAs (miRNAs) play essential roles in the progress of liver fibrosis by being involved in the differentiation, fat metabolism and ECM production of HSCs.

**Methods:**

The expression of miR-454 in LX-2 cells treated with TGF-β1 and in the fibrotic livers with *Schistosoma japonicum* infection was detected by qRT-PCR. The role of miR-454 on LX-2 cells was then analyzed by Western blot, flow cytometry and luciferase assay.

**Results:**

The results showed that the expression of miR-454 was down-regulated in the TGF-β1-treated LX-2 cells and miR-454 could inhibit the activation of HSCs by directly targeting Smad4. However, we found that miR-454 had no effect on cell cycle and cell proliferation in TGF-β1-treated LX-2. Besides these, miR-454 was found to be regulated in the process of *Schistosoma japonicum* infection.

**Conclusions:**

All the results suggested that miR-454 could provide a novel therapeutic approach for treating liver fibrosis, especially the liver fibrosis induced by *Schistosoma japonicum.*

## Background

Hepatic fibrosis, characterized by the excessive deposition of extracellular matrix (ECM), occurs in many types of liver diseases and reflects a balance between liver repair and scar formation [1]. Inflammation plays a key role in the development of liver fibrosis. During the progression of liver fibrosis, macrophages, lymphocytes, fibroblasts and other inflammatory cells can be stimulated by etiological factors [2]. Hepatic stellate cells (HSCs), which have been considered to be the major effector cells, can be activated by many inflammatory cytokines and undergo myofibroblastic transdifferentiation in the progress of liver fibrosis [1,3]. During this progress, the transforming growth factor-β1 (TGF-β1) is recognized as one of the main profibrogenic mediators [4,5] and plays a key role in the development of inflammation and subsequent liver fibrosis.

MicroRNAs (miRNAs) are endogenous, small and noncoding RNAs, which have the ability to regulate gene expression in a mature form by binding to the 3′-untranslated region (3′-UTR) of target mRNAs and repressing translation or inducing mRNA cleavage [6,7]. Previous studies have revealed that miRNAs play indispensable roles in the progress of liver fibrosis by being involved in the differentiation, fat metabolism and ECM production of HSCs [8,9] and the proliferation and apoptosis of HSCs [10-12]. Over-expression of miR-146a can suppress TGF-β1-induced HSC proliferation and induce HSC apoptosis [11]. miR-15b and miR-16 are essential for HSC apoptosis by targeting Bcl-2 through the caspase signaling pathway [12]. miR-335 can also inhibit HSC migration by decreasing the tenascin-C (TNC) expression [9]. Recently, the miR-454 family has been reported to be up-regulated in human colorectal cancer tissues and cell lines by targeting Smad4 [13]. However, there are no prior studies in which the effect of miR-454 on TGF-β1-induced HSC activation is considered. Therefore, in the present study, we attempted to observe the expression of miR-454 in activated HSCs and in the fibrotic livers, and then to study the role of miR-454 on HSC activation.

## Methods

### Animals and *Schistosoma japonicum (S. japonicum)* -infected liver fibrosis models

Healthy 4-6-wk-old male ICR mice were obtained from the Laboratory Animal Center of Nantong University. *S. japonicum* cercariae released from infected intermediate host snail *Oncomelania hupensis* were provided by the Jiangsu Institute of Parasitic Diseases (Wuxi, China). To construct the models infected with *S. japonicum*, mice were percutaneously infected with 20 ± 2 cercariae of *S. japonicum* and sacrificed on the 8^th^ week after infection. HE staining and sirius-red staining were performed to confirm that the liver fibrosis models were constructed successfully. Animal care and experimental procedures were approved by the Animal Ethics Committee of Nantong University.

### Cell culture and treatment

An immortalized human HSCs line, LX-2 cell line, was obtained from Xiang Ya Central Experiment Laboratory. LX-2 cells were cultured in Dulbecco’s modified Eagle’s medium (DMEM, Invitrogen, USA) supplemented with 10% fetal bovine serum (FBS, Invitrogen, USA) in a humidified incubator at 37°C with 5% CO_2_. LX-2 cells were plated in a 6-well plate and cultured for 24 h before transfection. Then mimics, inhibitors of miR-454 or the nonspecific (NS)-miRNA were transfected into the cells at a final concentration of 100 nmol/l using lipofectamine 2000 (Invitrogen, USA) according to the manufacturers instructions. The culture medium was discarded after transfection for 4-6 h and replaced with the fresh medium or the medium with TGF-β1 (Sigma, USA) at the concentration of 5 ng/ml for 48 h. The sequences of the miR-454 mimics, inhibitors and the NS-miRNA were all designed and synthesized by Genepharma Company in Shanghai, China.

### Construction and luciferase assay of 3′-UTR of Smad4

The wild-type and mutant sequences of the 3′-UTR of human Smad4 were amplified from LX-2 cells and cloned into the psi-CHECK-2 luciferase vector. For dual-luciferase reporter assays, the wild-type Luc-Smad4 or mutant Luc-Smad4 plasmids and miRNAs were co-transfected into LX-2 cells using lipofectamine 2000. After transfection for 48 h, the cells were collected and luciferase activity was analyzed by the dual-luciferase assay kit (Promega, USA).

### RNA isolation and quantitative real-time PCR (qRT-PCR)

Total RNA was isolated using the Trizol reagent (Invitrogen, USA) according to the manufacturer’s instruction and then reverse transcribed into cDNA using the Revert Aid First Strand cDNA Synthesis Kit (Thermo Fisher Scientific, USA). QRT-PCR was performed according to the protocol of SYBR Premix Ex Taq RT-PCR Kit (Takara, Japan) in the Eco Real-time PCR system (Illumina, USA). The miRNAs were extracted using RNAiso for Small RNA (Takara, Japan) and reverse transcribed for qRT-PCR using SYBR PrimeScript miRNA RT-PCR integrative kit (Takara, Japan) according to the manufacturer’s protocol. The sense primers for miRNA qRT-PCR were synthesized by Invitrogen (China) [10,13], and the universal anti-sense primer was obtained from Takara.

### Western blot

Proteins from LX-2 were extracted using RIPA lysis buffer (Beyotime, China) and quantified by the Bradford method (Sangon, China). Then the proteins were separated on 10% sodium dodecyl sulphate-polyacrylamide gel electrophoresis (SDS-PAGE) and electrotransferred onto polyvinylidene fluoride (PVDF) membranes. The membranes were blocked with 10% nonfat dry milk and then probed with primary antibodies against α-smooth muscle actin (α-SMA, Santa Cruz Biotechnology, USA), Smad4 (Santa Cruz Biotechnology, USA), PCNA (Abcam, USA) and glyceraldehyde phosphate dehydrogenase (GAPDH, Goodhere, China) at 4°C overnight. The membranes were then washed and incubated with horseradish peroxidase (HRP)-conjugated secondary antibodies. Then the membranes were visualized with ECL-chemiluminescent kit (Merck, Germany).

### MTT assay

The cell proliferation of LX-2 was determined using MTT assay. Firstly, the cells were plated at a density of 5 × 10^3^ cells/well in a 96-well culture plate for 24 h. After transfection with miR-454 mimics or NS-miRNA for 4-6 h, the culture medium was replaced with the fresh medium or the medium with TGF-β1. After LX-2 cells were pulsed with MTT (Sigma, USA) for 4 h, 100 μl of dimethylsulphoxide (DMSO) was added in the medium to dissolve the formazan products. The optical density (OD) was determined on an enzyme-linked immunosorbent assay (ELISA) reader (Bio-Tek) at 490 nm wavelength. All experiments were performed in triplicate and repeated at least three times.

### Flow cytometry

The cell cycle phase was determined by fluorescence activating cell sorter (FACS) analysis with propidium iodide (PI, Sigma, USA) staining. LX-2 cells were transfected with miR-454 mimics or NS-miRNA and treated with or without TGF-β1. After fixation, cells were treated with RNase A to digest RNA in the cells and stained with PI at 37°C for 30 min. The cells were then assessed by flow cytometry and the data were analyzed. All experiments were repeated at least three times.

### Statistical analysis

All experiments were performed in triplicate and the results were presented as mean ± SEM. The data were analyzed by One-Way ANOVA (LSD) using the SPSS 15.0 software to determine their significant differences. A value of *P*<0.05 was considered statistically significant.

## Results

### α-SMA is up-regulated in TGF-β1-treated LX-2 cells

**Figure 1:**
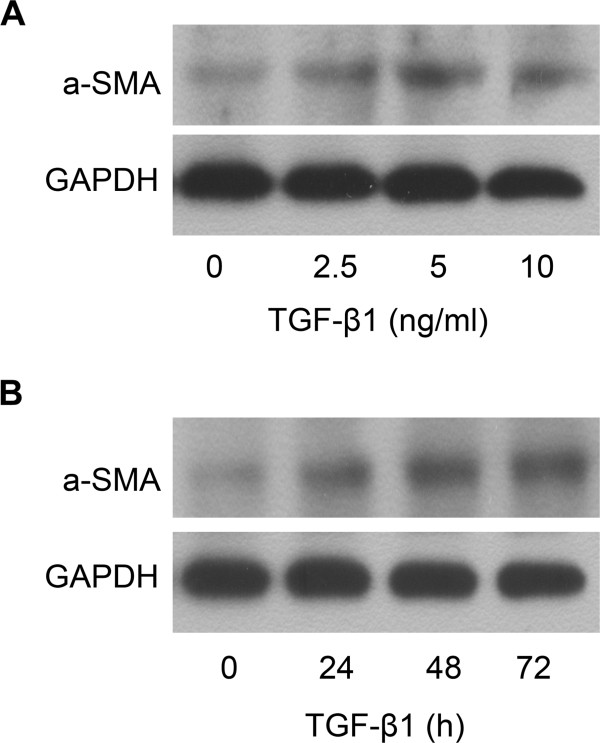
**TGF-β1 can up-regulate the expression of α-SMA in LX-2. (A)** Dose-dependent effect of TGF-β1 on α-SMA protein expression was analyzed by Western blot. **(B)** Time-dependent effect of TGF-β1 on α-SMA protein expression was analyzed by Western blot.

TGF-β1 is one of the most important molecules in activation of HSCs [14] and the expression of α-SMA, which is one of the characteristics of HSC activation, can be increased in HSCs treated with TGF-β1. Here, we showed that treatment of LX-2 cells with TGF-β1 at the concentration of 0, 2.5, 5 and 10 ng/ml for 48 h could result in the increased expression of α-SMA protein (Figure 1A). The expression of α-SMA protein in LX-2 cells was also increased in LX-2 cells treated with TGF-β1 (5 ng/ml) for 0, 24, 48 and 72 h (Figure 1B). The results showed that the expression of α-SMA protein in LX-2 cells could be up-regulated by TGF-β1 in the time- and dose-dependent manner.

### Expression of miR-454 is down-regulated in TGF-β1-treated LX-2 cells

**Figure 2:**
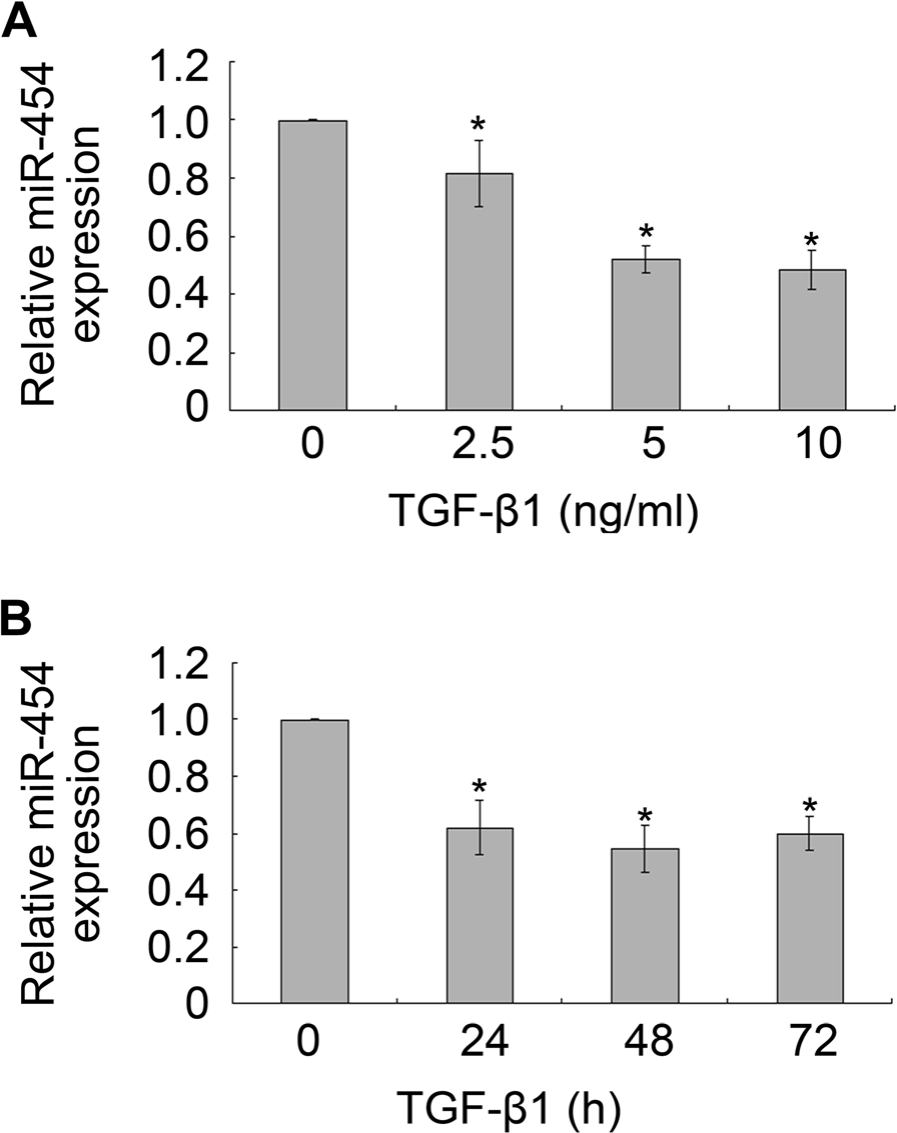
**The expression of miR-454 was down-regulated in TGF-β1-treated LX-2 cells. (A, B)** The expression of miR-454 in LX-2 cells which were treated with TGF-β1 at the indicated concentration or for the indicated time were detected by qRT-PCR. * *P* <0.05 vs control (cells with no stimulus).

The expression of miR-454 in TGF-β1-treated HSCs was evaluated by qRT-PCR. The data showed that the expression of miR-454 in LX-2 cells was also decreased when the cells were treated with TGF-β1 at the concentration of 2.5, 5 and 10 ng/ml for 48 h (Figure 2A), or when the cells were treated with TGF-β1 (5 ng/ml) for 24, 48 and 72 h (Figure 2B).

### Over-expression of miR-454 cannot regulate the proliferation and cell cycle distribution of LX-2 cells

**Figure 3:**
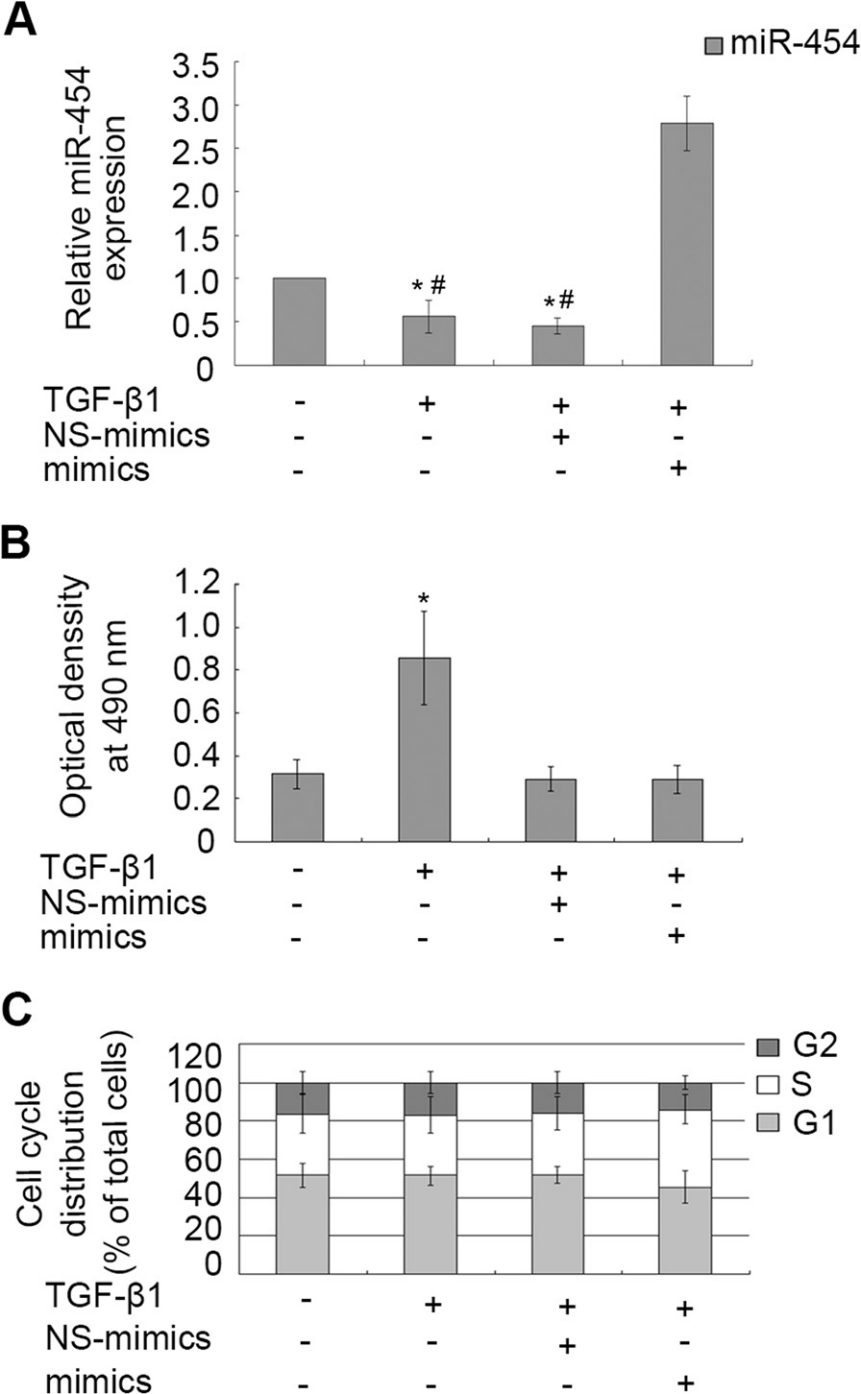
**The effect of miR-454 on proliferation and cell cycle distribution of TGF-β1-treated LX-2. (A)** The expression of miR-454 in LX-2 cells was analyzed by qRT-PCR. The expression of miR-454 could be down-regulated in the cells treated with TGF-β1 and in the cells co-treated with TGF-β1 and NS-miRNA. * *P* <0.05 vs control (cells with no stimulus). However, the down-regulation of miR-454 expression induced by TGF-β1 could be reversed in the cells transfected with miR-454 mimics (# *P* <0.05, compared with the group of the cells co-treated with TGF-β1 and miR-454 mimics). **(B)** The proliferation capacity of LX-2 cells was increased when the cells were treated with TGF-β1. * *P* <0.05 vs control (cells with no stimulus). However, miR-454 mimics could not regulate the proliferation of LX-2 cells by MTT assay (*P*>0.05, compared with the group of the cells co-treated with TGF-β1 and NS-miRNA). **(C)** The effect of miR-454 on cell cycle of LX-2 cells was analyzed by flow cytometry*. P*>0.05 vs control (cells with no stimulus)*.*

To investigate whether miR-454 was involved in the proliferation of HSCs, miR-454 mimics was transiently transfected into LX-2 cells and the results showed that the miR-454 expression was obviously increased in the cells with TGF-β1 and miR-454 mimics transfection, compared with the cells transfected with NS-mimics and TGF-β1 (Figure 3A). The down-regulation of miR-454 expression induced by TGF-β1 could be reversed in the cells transfected with miR-454 mimics, but not in the cells transfected with NS-miRNA (Figure 3A). Then, we analyzed whether the expression of miR-454 could affect cellular proliferation of LX-2 by MTT assay. The results showed that miR-454 had no effect on the proliferation of LX-2 with the existence of TGF-β1, compared with the group of the cells treated with TGF-β1 and NS-miRNA (*P*>0.05, Figure 3B). To further observe whether miR-454 could affect cell cycle in TGF-β1-treated LX-2 cells, we analyzed the effect of miR-454 on cell cycle of LX-2 cells by flow cytometry. We found that miR-454 mimics had no significant effect on cell cycle distribution, compared with those cells treated with TGF-β1 and NS-miRNA (*P*>0.05, Figure 3C). These data indicated that over-expression of miR-454 could not affect the proliferation and cell cycle distribution of TGF-β1-treated-LX-2 cells.

### Over-expression of miR-454 inhibits the expression of α-SMA in TGF-β1-treated LX-2 cells

**Figure 4:**
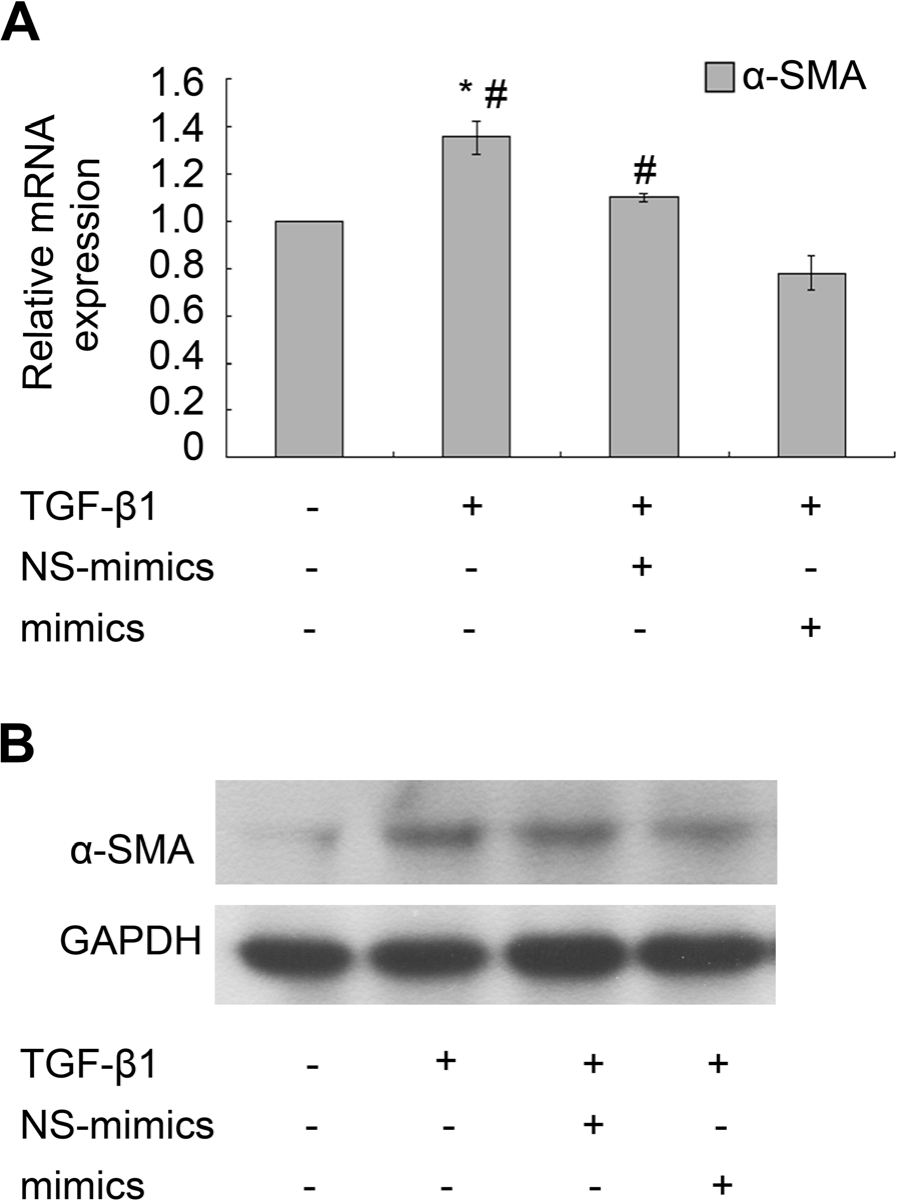
**miR-454 mimics could inhibit TGF-β1–induced α-SMA expression in LX-2 cells. (A)** miR-454 mimics reduced the levels of α-SMA mRNA expression in TGF-β1-treated LX-2 cells as detected by qRT-PCR. * *P* <0.05 vs control (cells with no stimulus). # *P* <0.05 vs the group of TGF-β1 + mimics+. **(B)** Western blot analysis confirmed that α-SMA protein expression was inhibited by miR-454 mimics in TGF-β1-treated LX-2 cells.

As shown in the results in Figure 4, the expression of α-SMA mRNA (Figure 4A) and protein (Figure 4B) in TGF-β1-treated LX-2 cells were both increased significantly. The expression of α-SMA mRNA (Figure 4A) and protein (Figure 4B) were all inhibited obviously in TGF-β1-treated LX-2 cells transfected with miR-454 mimics, compared with those cells transfected by NS-miRNA and co-treated with TGF-β1. The data demonstrated that over-expression of miR-454 significantly decreased the expression of α-SMA. miR-454 may be involved in the TGF-β1-induced HSC activation.

### Smad4 is a direct target of miR-454 in HSCs

**Figure 5:**
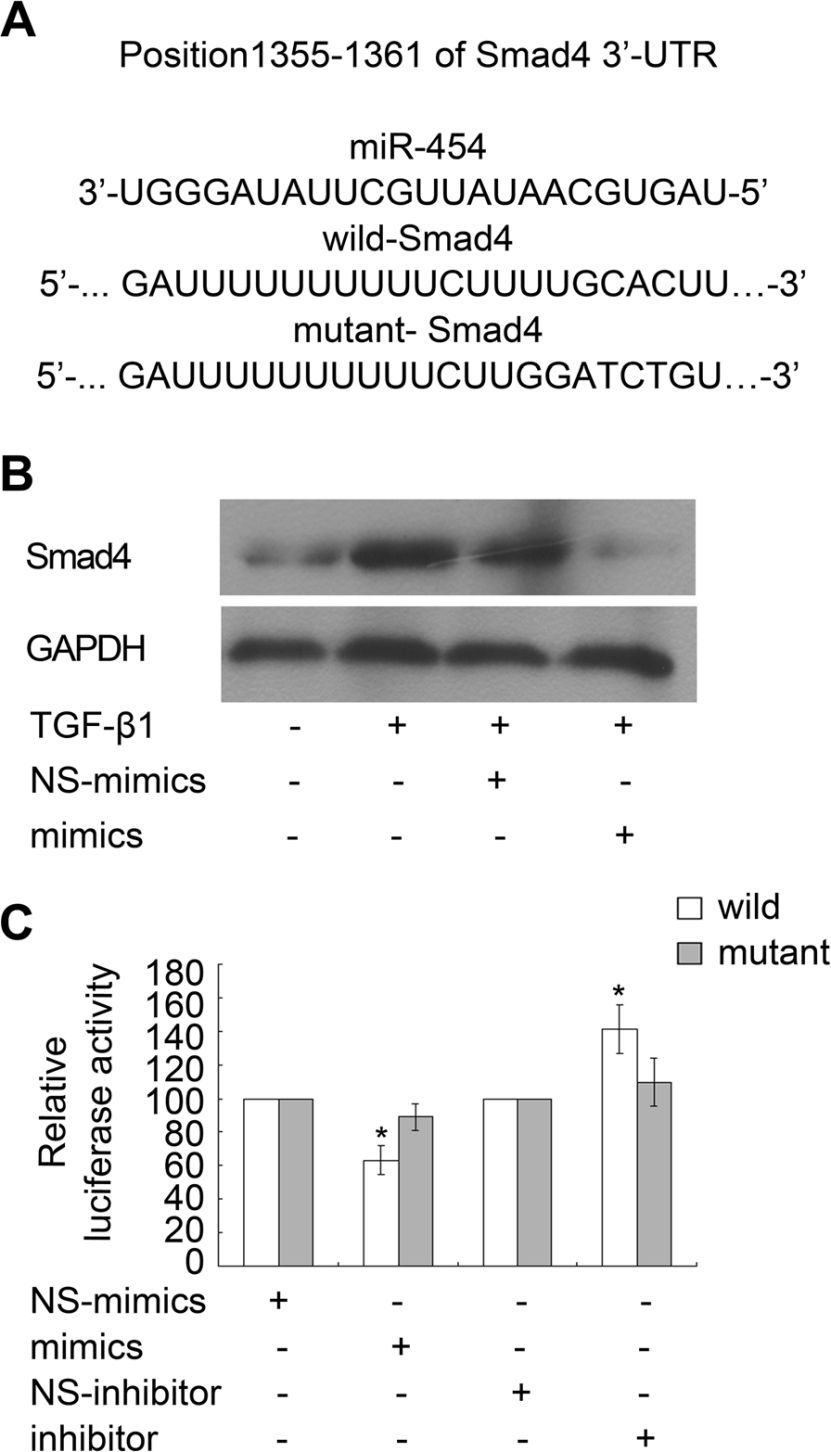
**miR-454 directly targeted Smad4 in LX-2 cells. (A)** Sequence alignment of putative miR-454-binding site in the 3′-UTR of Smad4 and the sequence of the mutant 3′-UTR of Smad4 were shown. **(B)** The expression of Smad4 protein was reduced by miR-454 in TGF-β1-treated LX-2 cells,as determined by Western blot. **(C)** Over-expression of miR-454 could induce the decrease of the luciferase activity of the wild-type Luc-Smad4 reporter, but not that of the mutant-type Luc-Smad4 reporter. Meanwhile, miR-454 inhibitor could increase luciferase activity of the wild-type 3′-UTR of Smad4. * *P* <0.05 vs one’s own control group (cells transfected with NS-miRNA and wild or mutant plasmids).

Potential targets of miR-454 were then predicted using miRbase Targets, miRanda and TargetScan 5 platform, and Smad4 is the most attractive target of miR-454 for further study (Figure 5A). Smad4 is a critical participator in the regulation of TGF-β1 signaling and it has been reported to be a target of miR-454 in colorectal cancer cells [13]. To determine whether miR-454 could also regulate the expression of Smad4 in HSCs, miR-454 mimics and NS-miRNA were transfected into TGF-β1-treated LX-2 cells, respectively. The results revealed that the expression of Smad4 protein in LX-2 cells was up-regulated in response to TGF-β1 stimulation and this effect could be inhibited by the mimics of miR-454 (Figure 5B).

Furthermore, in order to investigate whether Smad4 was directly targeted by miR-454 expression, we constructed the luciferase reporter plasmids containing wild-type or mutant 3′-UTR sequences of Smad4. The data showed that the luciferase reporter activity of the reporter plasmids containing the wild-type 3′-UTR of Smad4, but not the mutant 3′-UTR of Smad4, could be reduced by miR-454 mimics and increased by miR-454 inhibitor (Figure 5C). Therefore, miR-454 could inhibit TGF-β1-induced LX-2 activation by the suppression of Smad4 expression.

### Expression of miR-454 is down-regulated in *S. japonicum*-induced liver fibrosis models

**Figure 6:**
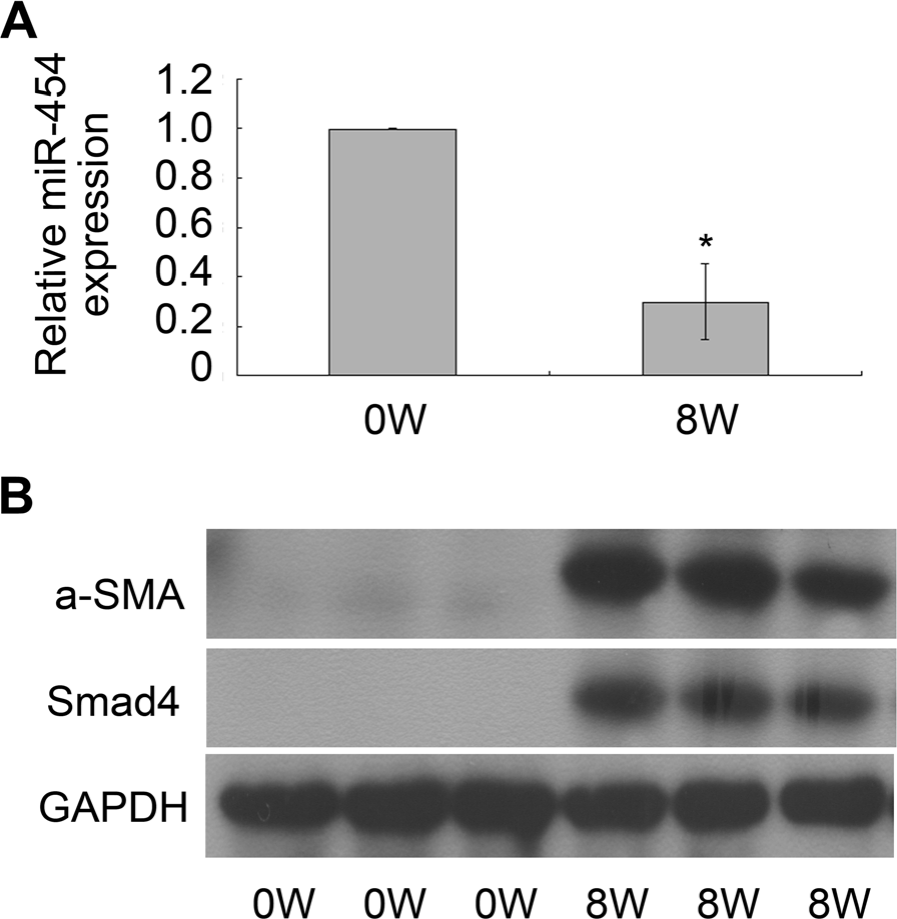
**miR-454 was down-regulated in *S. japonicum*-infected fibrotic livers. (A)** The expression of the miR-454 was down-regulated in the fibrotic tissues infected with *S. japonicum* for 8 weeks as detected by qRT-PCR. * *P* <0.05 vs control (normal mice). **(B)** The levels of α-SMA and Smad4 expression were all up-regulated in the fibrotic livers infected with *S. japonicum* for 8 weeks as determined by Western blot.

In addition, we also investigated the expression of miR-454 in mouse models with liver fibrosis induced by *S. japonicum*. QRT-PCR was performed to analyze the relative expression level of miR-454 in fibrotic livers. The data clearly indicated that miR-454 was sharply declined in the livers infected with *S. japonicum* for 8 weeks (Figure 6A). Meanwhile, the results also revealed that the expression of α-SMA and Smad4 were up-regulated significantly in the fibrotic livers at 8 weeks post-infection, compared with the normal livers (Figure 6B). Therefore, the results suggested that miR-454 may play an important role on *S. japonicum-*infected liver fibrosis.

## Discussion

Liver fibrosis, which is characterized by an accumulation of ECM, is a common response to many liver injuries. During the progression of liver fibrosis, quiescent HSCs, which can be provoked by hepatitis B and C viruses, nonalcoholic steatohepatitis, alcoholism and schistosome infection, can activate into a myofibroblast-like phenotype [2,4]. Recently, some studies have focused on the functions of miRNAs on liver fibrosis [8,9,15]. Previous studies have shown that miR-181b expression could be increased in HSC-T6 cells treated with TGF-β1 and miR-181b mimics could significantly promote the proliferation of HSC-T6 cells by directly targeting p27 [8]. The expression of miR-150 and miR-194 were both down-regulated in the fibrotic livers of rats induced by bile duct-ligation (BDL), while over-expression of miR-150 and miR-194 could inhibit the proliferation of LX-2 cells [15]. In addition, miR-21 significantly activated hepatic stellate cells through the PTEN/Akt pathway [16]. Consistent with the results obtained by Ji *et al.*[10] that the expression of miR-454 was depressed in activated rat HSCs, we found that the expression of miR-454 was down-regulated in LX-2 cells treated with TGF-β1.

TGF-β1/Smads signaling pathway is a key mediator to induce HSC activation and liver fibrosis [17]. TGF-β1 can promote the formation of ECM through stimulation of the synthesis and secretion of Type-1 or Type-2 collagen and α-SMA [18,19]. In addition, previous studies have indicated that anti-sense Smad4 gene could block TGF-β1 signal transduction by reducing the expression of Smad4 to inhibit the production of ECM and ameliorate hepatic fibrosis [20-22]. In this research, we also found that TGF-β1 could induce the activation of HSCs and reduce the expression of miR-454. Besides these, over-expression of miR-454 could inhibit the expression of α-SMA induced by TGF-β1 stimulation. However, miR-454 did not affect the proliferation and cell cycle in TGF-β1-treated LX-2 cells. Since previous studies have shown that the expression of miR-454 was significantly up-regulated in colon cancer and in HCT116 cells, and miR-454 contributed to colon tumorigenesis by directly repressing the expression of Smad4 [13], we further investigated whether Smad4 was also a direct target of miR-454 to regulate the expression of α-SMA in TGF-β1-treated LX-2 cells. Our experiments demonstrated that miR-454 could influence the level of Smad4 expression in TGF-β1-treated LX-2 cells, and over-expression of miR-454 could inhibit the luciferase activity of the wild-type Luc-Smad4 plasmid, but not the mutant-type Luc-Smad4 plasmid. Meanwhile, miR-454 inhibitor could increase luciferase activity of the wild-type 3′-UTR of Smad4 in LX-2 cells. These data indicated that miR-454 may down-regulate the expression of α-SMA and therefore inhibit the activation of TGF-β1-treated HSCs by directly targeting Smad4. However, since previous researches have found that over-expression of miR-146a could also inhibit TGF-β1-induced HSC proliferation by targeting Smad4 [11], we cannot rule out the possibility that other miRNAs could participate in the modulation of HSC activation and proliferation. We also cannot rule out the possibility that miR-454 could inhibit HSC activation through other signaling pathways, since multiple mRNAs are predicted by some softwares to be targeted by the same miRNA. Hence, further studies are needed to reveal the complex relationship among miRNAs (miR-146a and others), targets and hepatic fibrosis.

Schistosomiasis, which is caused by schistosomes, is one of the most common causes of hepatic fibrosis. Granulomatous inflammation is an initial characteristic manifestation of liver fibrosis induced by schistosomes. Recently, some studies have reported that the expression of some miRNAs can be regulated in the mouse liver infected with *S. japonicum*, and it suggests that miRNAs may be involved in the process of inflammatory granuloma formation and the subsequent hepatic fibrosis [23,24]. Meanwhile, studies have also focused on the expression of miRNA profiles in the host liver infected with schistosomes to understand the molecular mechanisms of schistosomal hepatopathy, which differs from other chronic hepatopathy [25]. In this study, we also observed the expression of miR-454 in the mouse liver during *S. japonicum* infection. The results showed that the level of the miR-454 was down-regulated in the fibrotic livers infected with *S. japonicum* for 8 weeks. On the contrary, the levels of α-SMA and Smad4 expression were all up-regulated. Since the tendency of the expression of Smad4 was opposite to that of miR-454 in the process of hepatic fibrosis induced by *S. japonicum* infection, we hypothesized that miR-454 may be involved in the progression of liver fibrosis induced by *S. japonicum* infection through the TGF-β1/Smad4 pathway.

## Conclusions

In summary, our results indicated that miR-454 could inhibit the activation of HSCs by directly targeting Smad4. Moreover, miR-454 was regulated in the process of hepatic fibrosis induced by *S. japonicum* infection. Thus, miR-454 may provide a novel therapeutic approach for treating liver fibrosis, especially the liver fibrosis induced by *S. japonicum.*

## Competing interests

The authors declare that they have no competing interests.

## Authors’ contributions

YND and DDZ conceived and designed the study. DDZ, XH and FFX performed the experiments. DDZ, JLC, XJW and LBZ constructed the animal models. DDZ, XH and YND analyzed the data. XLS, JRF, WS and HYQ provided logistical and scientific support for the study and assisted in writing the manuscript. DDZ, XH and YND drafted the manuscript. All authors have read and approved the final manuscript.
